# The Role of Combining Probiotics in Preventing and Controlling Inflammation: A Focus on the Anti-Inflammatory and Immunomodulatory Effects of Probiotics in an *In Vitro* Model of IBD

**DOI:** 10.1155/2022/2045572

**Published:** 2022-11-08

**Authors:** Shadi Aghamohammad, Amin Sepehr, Seyedeh Tina Miri, Saeideh Najafi, Mohammad R. Pourshafie, Mahdi Rohani

**Affiliations:** ^1^Department of Bacteriology, Pasteur Institute of Iran, Tehran, Iran; ^2^Department of Biology, Science and Research Branch Islamic Azad University, Tehran, Iran

## Abstract

**Objective:**

IBD is an inflammatory disease with abnormalities such as dysbiosis and abnormal immune system activity. Probiotics, as live beneficial microorganisms, play a role in maintaining health through various mechanisms, including the modulation of the immune system and the control of inflammation. Here, we aimed to investigate the efficacy of a probiotic mixture of *Lactobacillus* spp. and *Bifidobacterium* spp. in modulating JAK/STAT and NF-kB inflammatory signaling pathways.

**Method:**

A quantitative real-time polymerase chain reaction (qPCR) assay was conducted to analyze the expression of JAK/STAT and inflammatory genes after treatment with the probiotic mixture before, after, and simultaneously with the sonicated pathogen in the HT-29 cell line. The production of IL-6 and IL-1*β* after probiotic treatment was investigated via cytokine assay.

**Results:**

Treatment with probiotics resulted in downregulation of *TIRAP*, *IRAK4*, *NEMO*, and *RIP* genes in the NF-kB pathway and *JAK*/*STAT* genes compared with sonicat-treated cells as inflammation inducers. The production of IL-6 and IL-1 decreased after probiotic treatment.

**Conclusions:**

The probiotic mixture of *Lactobacillus* spp. and *Bifidobacterium* spp. showed anti-inflammatory effects by modulating JAK/STAT and NF-kB signaling pathways. The use of probiotics could be considered as an appropriate complementary treatment for patients with inflammatory bowel disease.

## 1. Introduction

Probiotics as live beneficial microorganisms have various roles in maintaining health. Prevention of gastrointestinal symptoms such as diarrhea, lowering cholesterol levels, and prevention of cancer and allergy are some of the notable effects of probiotics [1]. In addition, one of the most important remarkable effects of probiotics is to influence the consequences of dysbiosis. An imbalance of the intestinal microbiome can lead to an increase in pathogenic bacteria and a decrease in the healthy microbiota. This process eventually leads to abnormal function of immune cells, including Th1 and Th17 [2]. Th1 and Th17 could influence the inflammatory response via the production of IL-2 and IL-17. However, the abnormal activity of these cells usually leads to chronic inflammation [3]. Probiotics could influence this process via immunomodulatory functions. Indeed, Tregs suppress the immune response and prevent inflammation in the colon by producing IL-10. Probiotics could modulate the immune system and reduce inflammatory conditions by causing an effect on Tregs [4]. *Lactobacillus* spp. and *Bifidobacterium* spp. are two probiotic members that have anti-inflammatory effects. The anti-inflammatory characteristics of these probiotic strains, especially in the intestinal epithelial cells, and the reduction of symptoms in patients with ulcerative colitis have been demonstrated [5]. Also, various studies have been demonstrated that using the combination of probiotic strains compared with individual strains has stronger effects on improving health conditions [6].

One of the inflammatory diseases that pose challenges to patients is inflammatory bowel disease (IBD). Ulcerative colitis (UC) and Crohn's disease (CD) are two subtypes of IBD with some differences, including the location of the GI tract that is affected. The exact etiology of IBD is not yet fully understood. However, risk factors include genetics, stress, antibiotic use, dysbiosis of the gut microbiome, and abnormal immune system function [7]. IBD is described as a disease with remission and relapse phases. The exact timing of relapse is not known, but the use of an agent or strategy that could prolong the duration of the remission phase would be crucial for patients with IBD [8]. As mentioned earlier, probiotics have immunomodulatory effects, and since IBD may be caused by immunologic imbalance, probiotic intake may be suitable to control IBD. The immunomodulatory properties of probiotics could be different. Various molecular signaling pathways, for instance, could be affected by probiotics, so probiotics exert their immunoregulatory and anti-inflammatory properties [9]. The Janus kinase/signal transducer and activator of transcription (JAK/STAT) and the nuclear factor kappa-light-chain-enhancer of activated B Cells (NF-kB) are two signaling pathways involved in various human diseases, including inflammatory disorders. The association of these signaling pathways with pre- and anti-inflammatory cytokines may elucidate the link with inflammatory diseases such as IBD. Since inflammatory cytokines play an important role in the progression of IBD, finding an agent with positive modulatory effects on the aforementioned signaling pathways could be useful for the treatment or prevention of IBD [10, 11].

Our laboratory has demonstrated the phenotypic effects of probiotics on the control of inflammatory status [12]. Also, the immunomodulatory and anti-inflammatory effects of different probiotic strains before inflammation induction have been shown to clarify the preventive effects of probiotic strains to reduce inflammation [13]. Indeed, the identification of the exact molecular effects of probiotics on signaling pathways involved in the progression of inflammation may clarify the beneficial effects of probiotics. In the present study, we aimed to demonstrate the immunomodulatory and anti-inflammatory effects of *Lactobacillus* spp. and *Bifidobacterium* spp. as a mixture before, after, and during inflammation by causing an effect on JAK/STAT and NF-kB signaling pathways to determine whether they could be used as therapeutic or preventive options for inflammatory conditions, including IBD.

## 2. Material and Methods

### 2.1. Bacterial Strain, Culture Medium, and Growth Conditions

Here, the in vitro assay was conducted to evaluate the effects of probiotics on NF-kB and JAK/STAT signaling pathways in the HT-29 cell line. Four *Lactobacillus* spp. including *L. plantarum*, *L. rhamnosus*, *L. brevis*, and *L. reuteri* and three *Bifidobacterium* spp. including *B. bifidum*, *B. longum*, and *B. infantis* were isolated from stool samples and breast milk as previously reported [12, 14]. The preparation of probiotic cocktails in addition to pathogenic bacteria, including enterotoxin-producing *Escherichia coli* (ETEC) and *Salmonella typhimurium* (ST) were explained [13]. The experimental protocols were established following the Declaration of Helsinki and approved by the ethics committee of Pasteur Institute of Iran (IR.PII.REC.1398.060). All methods were carried out in accordance with relevant guidelines and regulations.

### 2.2. Treatment of HT-29 Cells with Probiotics

HT-29 cells were exposed to various bacteria, including sonicated pathogenic enterotoxigenic *E. coli* (SP-ETEC), sonicated pathogen *Salmonella typhi*, and *Lactobacillus*/*Bifidobacterium* spp. mixture.

#### 2.2.1. Investigation of the Preventive Effects of *Lactobacillus/Bifidobacterium* Mixture

To study the effect of probiotics before inflammation induction, the *Lactobacillus/Bifidobacterium* mixture was first added to HT-29 cell line, and after 6 hours, SP-ETEC and SP-*Salmonella typhi* were added to induce inflammation.

#### 2.2.2. Investigation of the Therapeutic Effects of *Lactobacillus/Bifidobacterium* Mixture at the Beginning of Inflammation

To show the efficacy of *Lactobacillus*/*Bifidobacterium* at the beginning of inflammation induction *Lactobacillus*/*Bifidobacterium* and sonicated pathogens were added to the HT-29 cell line simultaneously.

#### 2.2.3. Investigation of the Therapeutic Effects of Lactobacillus/Bifidobacterium Mixture after Inflammation Induction

To study the effect of *Lactobacillus/Bifidobacterium* mixture after inflammation induction, SP-ETEC and SP-*Salmonella typhi* were first added to the HT-29 cell line, and *Lactobacillus*/*Bifidobacterium* was added after 6 hours to determine the presumed effects. Each treatment was performed in two separate biological replicates and three separate technical replicates.

In the next step, each well was washed twice with PBS to remove the nonadherent bacteria. These treatments were performed in duplicate and cell culture was maintained at 37°C and 5% CO_2_ for up to 48 hours. The determination of MOI was performed as previously described [15].

### 2.3. Cytokine Assays

To measure the production of proinflammatory cytokines, including IL-6 and IL-1*β*, the cell culture supernatant was centrifuged at 6000 rpm, the sediment was discarded, and the supernatant was collected to determine using an ELISA kit (Karmanian Pars Gene, Iran) according to the manufacturer's protocols.

### 2.4. RT-PCR of Inflammatory Signaling Pathway Genes

Total RNA was extracted according to the manufacturer's instructions (Roche, Germany). The cDNA template was synthesized with the cDNA synthesis kit (Yekta Tajhiz, Iran) according to the manufacturer's instructions. The online PrimerBank website (http://pga.mgh.harvard.edu/primerbank) was used to choose the qPCR primers (Table 1). All the primers were tested using gradient PCR to get an appropriate annealing temperature. The mRNA quantification of studied genes was evaluated with the ABI step one plus detection system (Applied Biosystems, USA co) using SYBR Green master mix (Amplicon Bio, Denmark). All the reactions were performed in duplicate. The formula RQ = 2^−ΔΔCt^ was used to get relative gene expression in the comparative CT method [16]. The appropriate internal control gene, glyceraldehyde 3-phosphate dehydrogenase (*gapdh*), was selected as a housekeeping gene to normalize the data.

### 2.5. Statistical Analysis

Graphs and statistical analyses of the data were performed using GraphPad Prism 8 software to compare the variables of the different groups. Statistical differences between different groups, including control (C), sonicated pathogen (SP), the first administration of *Lactobacillus/Bifidobacterium* and second administration of sonicated pathogen (LBP), the first administration of sonicated pathogen and the second administration of *Lactobacillus/Bifidobacterium* (PLB), and the concurrent administration of *Lactobacillus/Bifidobacterium* and sonicated pathogen (*P* + LB), were determined using ANOVA. *Pvalues* < 0.05 were considered statistically significant. Results were expressed as standard deviation (SD).

## 3. Results

The efficacy of probiotics in up- or down-regulation of *JAK/STAT* and inflammatory genes was investigated by comparing probiotic-treated HT-29 cells with negative control cells (unexposed HT-29 cells) and HT-29 cells exposed to the sonicated pathogen as the positive control. It should be said that the concept of treatment phases refers to *Lactobacillus/Bifidobacterium* treatments before, after, and during the induction of inflammation.

### 3.1. *Lactobacillus/Bifidobacterium* Could Generally Decrease the Expression Level of *STAT* Genes

**Figure 1:**
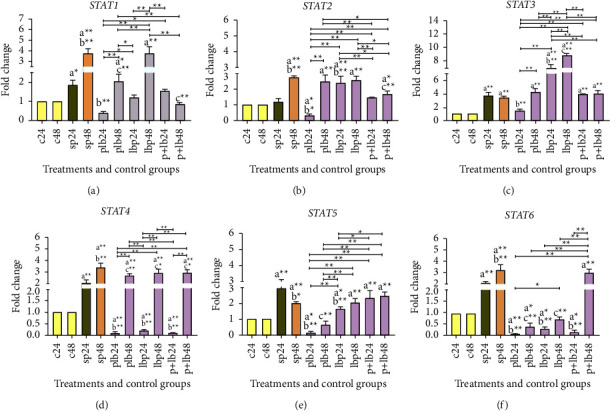
Relative gene expression (mean fold change) of (a) *STAT1*, (b) *STAT2*, (c) *STAT3*, (d) *STAT4*, (e) *STAT5*, and (f) *STAT6* in the different groups of treatments. Data were normalized with *GAPDH*. Data were represented as mean SD. The number 24 and 48 refers to different time orders of HT-29 cell line treatments. (C) represents control; (P) represents pathogen; PLB represents first pathogen and then Lactobacillus/Bifidobacterium; LBP represents first Lactobacillus/Bifidobacterium and then sonicated pathogen; P + LB represents sonicated pathogen and then *Lactobacillus/Bifidobacterium* added simultaneously. Data were considered statistically significant when *p* < 0.05 (^*∗*^*p* < 0.05, ^*∗*^^*∗*^*p* < 0.001). Letter (a) indicates the relatedness between C24 and C48 with other treatments, letter (b) shows the relatedness between sp24 and other treatments, and letter (c) shows the relatedness between sp48 with other treatments. The relatedness between other treatments is shown with brackets.

Gene expression data from *STAT* are shown in Figure 1. The expression level increased after treatment with sonicated pathogens (*p* < 0.05) compared to negative controls.

The comparative analysis of *STAT1* gene expression showed that the overall trend was downward in most treatment phases, especially compared to the sonicated pathogens after 48 hours of treatment (SP48). *Lactobacillus/Bifidobacterium* treatment after inflammation induction in the 24 hours of treatment (PLB24) had almost the strongest reduction effect (*p* < 0.05). Treatment with our probiotic mixture before inflammation induction (LBP48) had the least reduction effect. It could be said that the probiotics could have a better reducing effect on the post-treatment status.

In *STAT2*, the results were similar to those in *STAT1*. Again, *Lactobacillus/Bifidobacterium* treatment had the strongest reduction effect after inflammation induction in the 24 hours of treatment (PLB24) (*p* < 0.001). *Lactobacillus/Bifidobacterium* treatment with concomitant inflammation induction (*P* + LB48) also had a remarkable reduction effect (*p* < 0.05). Again, using our probiotic mixture before inflammation induction (LBP24 and LBP48) had the least reduction effect. Therefore, the post-treatment had the better reduction effect.

In *STAT3*, results showed that using *Lactobacillus/Bifidobacterium* before inflammation induction (LBP24 and LBP48) could increase the expression level (*p* < 0.001). Again, using *Lactobacillus/Bifidobacterium* after inflammation induction (PLB24) had the greatest reduction effect (*p* < 0.001) and using probiotic mixture before inflammation induction has the least reduction effects.

The comparative analysis of *STAT4* gene expression showed that the general trend was downward, especially in the first 24 hours of treatment. *Lactobacillus/Bifidobacterium* was able to significantly decrease the expression level in all three treatment phases (*p* < 0.05).

For *STAT5*, the addition *of Lactobacillus/Bifidobacterium* before and after inflammation induction (LBP24 and PLB24) had significant effects on reducing expression levels compared to the positive control in the first 24 hours of treatment (SP24) (*p* < 0.01). It should be noted that using *Lactobacillus/Bifidobacterium* after inflammation induction (PLB24 and PLB48) had a reducing effect at both time orders. It has been fully shown that the use of probiotic strains in post-treatment had a better reduction effect.

The comparative analysis of *STAT6* gene expression showed that the general trend was downward in most treatment phases. All treatments with *Lactobacillus/Bifidobacterium* were able to decrease the expression level, except for *P* + LB48. The addition of *Lactobacillus/Bifidobacterium* before and after inflammation induction at both time points (LBP24 and 48, PLB24 and 48) had a reducing effect. The use of *Lactobacillus/Bifidobacterium* after inflammation induction in the first 24 hours of treatment (PLB24) reduced the expression level to almost zero.

Regarding the expression of *STAT* genes, it can be said that post-treatment with probiotic strains had the best reduction effects on all six *STAT* genes. This effect could show the suitable therapeutic property of probiotics, which make these strains suitable as complementary treatments.

### 3.2. Significant Reduction Effects Could Be Seen via *Lactobacillus/Bifidobacterium* Treatments on *JAK* Genes

**Figure 2:**
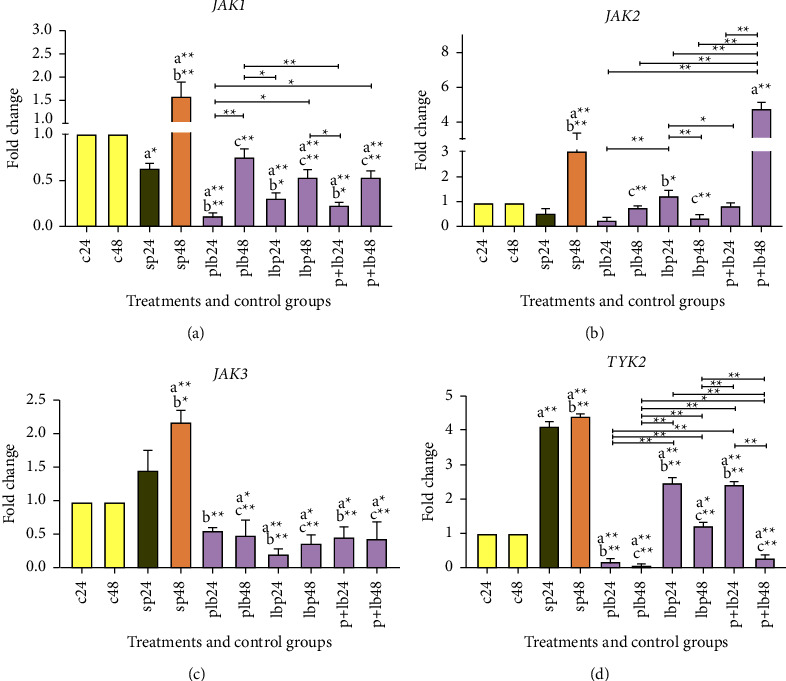
Relative gene expression (mean fold change) of (a) *JAK1*, (b) *JAK2*, (c) *JAK3*, (d), and (e) *TYK2* in the different groups of treatments. Data were normalized with *GAPDH*. Data were represented as mean SD. The number 24 and 48 refers to different time orders of HT-29 cell line treatments. (C) represents control; (P) represents pathogen; PLB represents first pathogen and then *Lactobacillus/Bifidobacterium*; LBP represents first *Lactobacillus/Bifidobacterium* and then sonicated pathogen; P + LB represents sonicated pathogen and then *Lactobacillus/Bifidobacterium* added simultaneously. Data were considered statistically significant when *p* < 0.05 (^*∗*^*p* < 0.05, ^*∗*^^*∗*^*p* < 0.001). Letter (a) indicates the relatedness between C24 and C48 with other treatments, letter (b) shows the relatedness between sp24 and other treatments, and letter (c) shows the relatedness between sp48 with other treatments. The relatedness between other treatments is shown with brackets.

Expression data from *JAK* are shown in Figure 2. The comparative analysis of *JAK* genes expression between positive and negative controls showed that the sonicated pathogens could significantly increase gene expression, especially after 48 hours of treatment (*p* < 0.001). A downward trend in gene expression was observed for all *JAK* genes, including *JAK1*, *JAK2*, *JAK3*, and *TYK2*.

In *JAK1*, *Lactobacillus/Bifidobacterium* treatment had the strongest reduction effect after inflammation induction in the 24 hours of treatment (PLB24) (*p* < 0.001). For *JAK2*, almost all treatments were able to down-regulate the expression level, except for LBP24. All treatments could have reduction effects in the expression level of *JAK3.* In *TYK2*, the same result (the reduction effect) could be seen in all treatments. It should be noted that the expression level in PLB48 decreased to zero (see Figure 2(d)). As with *STAT* genes, post-treatment with our probiotic strains could reduce the expression of *JAK* genes (especially in JAK1 and TYK2).

### 3.3. *Lactobacillus/Bifidobacterium* Treatment Reduces the Expression of Inflammatory Genes

**Figure 3:**
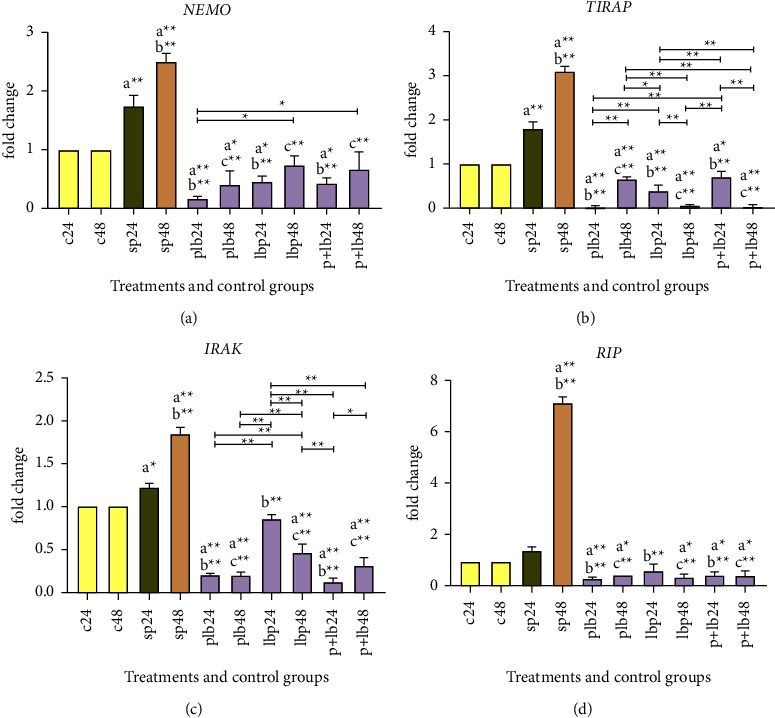
Relative gene expression (mean fold change) of (a) *NEMO*, (b) *TIRAP*, (c) *IRAK*, (d) *RIP* in the different groups of treatments. Data were normalized with *GAPDH*. Data were represented as mean SD. The number 24 and 48 refers to different time orders of HT-29 cell line treatments. (C) represents control; (P) represents pathogen; PLB represents first pathogen and then *Lactobacillus/Bifidobacterium*; LBP represents first *Lactobacillus/Bifidobacterium* and then sonicated pathogen; P + LB, sonicated pathogen and then *Lactobacillus/Bifidobacterium* added simultaneously. Data were considered statistically significant when *p* < 0.05 (^*∗*^*p* < 0.05, ^*∗*^^*∗*^*p* < 0.001). Letter (a) indicates the relatedness between C24 and C48 with other treatments, letter (b) shows the relatedness between sp24 and other treatments, and letter (c) shows the relatedness between sp48 with other treatments. The relatedness between other treatments is shown with brackets.

Inflammatory gene expression data are shown in the comparative analysis of inflammatory gene expression, including *NEMO*, *TIRAP*, *IRAK*, and *RIP* between the positive and negative controls which showed that sonicated pathogens could significantly increase gene expression, especially after 48 hours (*p* < 0.001). The expression level in some genes, including *TIRAP* using *Lactobacillus/Bifidobacterium* in all three treatment phases, could decrease the expression level to near zero (see Figure 3(b)). Although, as can be seen in Figure 3, all treatments were able to reduce the expression level of inflammatory genes, a closer look at the magnitude of the reduction shows that post-treatment had a greater impact on the reduction (especially for the genes *NEMO*, *TIRAP*, and *RIP*). Again, the expression of inflammatory genes could show the complementary therapeutic effect of our probiotic strains.

### 3.4. The Results of Cytokine Production

**Figure 4:**
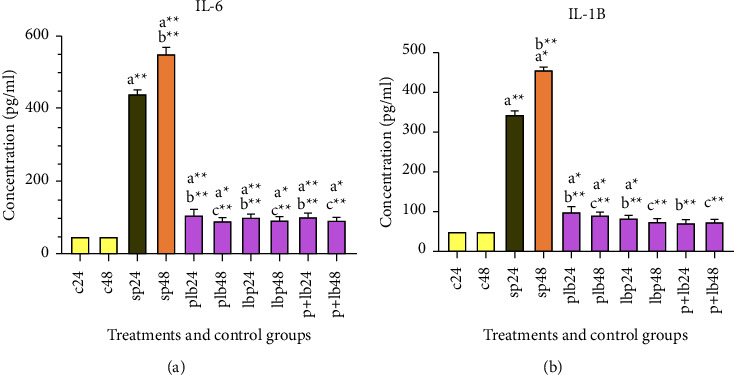
Different levels of concentrations of (a) IL-6 and (b) IL-1*β*. Data were represented as mean SD. The number 24 and 48 refers to different time orders of HT-29 cell line treatments. (C) represents control; (P) represents pathogen; PLB represents first pathogen and then *Lactobacillus/Bifidobacterium*; LBP represents first *Lactobacillus/Bifidobacterium* and then sonicated pathogen; **P** **+** **LB** represents sonicated pathogen and then *Lactobacillus/Bifidobacterium* were added simultaneously. Data were considered statistically significant when *p* < 0.05 (^*∗*^*p* < 0.05, ^*∗*^^*∗*^*p* < 0.001). Letter (a) indicates the relatedness between C24 and C48 with other treatments, letter (b) shows the relatedness between sp24 and other treatments, and letter (c) shows the relatedness between sp48 with other treatments. The relatedness between other treatments is shown with brackets.

The results of pro-inflammatory cytokine production could be seen in Figure 4. Cytokine production was significantly higher after treatment with SP. However, *Lactobacillus/Bifidobacterium* treatment significantly decreased cytokine production before, after, and during inflammation. No significant difference was seen between the different treatment phases.

## 4. Discussion

IBD as a chronic inflammatory disease has different phases of relapses and remissions. Patients usually present with various symptoms, including diarrhea, abdominal pain, and bloody stools, as well as the production of inflammatory cytokines [17]. As mentioned above, abnormal responses of the innate and adaptive immune systems are one of the causative processes involved in IBD. The inhibition of Treg function is a process that could occur in the pathogenesis of IBD. Treg cells are associated with anti-inflammatory cytokines such as IL-10 and therefore have an inhibitory effect on other Th cells including Th1 and Th17. Inhibition of Tregs could lead to overproduction of pro-inflammatory cytokines. The other process that may be involved in the pathogenesis of IBD is the activation of the NF-kB pathway, which is associated with IL-17, and the production of pro-inflammatory cytokines, including IL-6 [18]. JAK/STAT is another signaling pathway associated with pro-inflammatory and anti-inflammatory cytokines and therefore may play a crucial role in the pathogenesis of IBD [19]. The use of an agent, such as probiotics, that could affect these signaling pathways might be suitable to treat or prevent the symptoms of IBD. As mentioned above, the combination of probiotics has better effects on health status compared to individual strains [6]. Therefore, we aimed to use probiotics (Lactobacillus/Bifidobacterium) before, after, and during inflammation induction and evaluate the molecular effects of these strains to determine whether probiotics have anti-inflammatory effects as preventive and/or therapeutic agents.

The results of our previous in vivo study showed anti-inflammatory effects of probiotics [12]. In the present study, we performed in vitro molecular studies to investigate the exact mechanisms of the anti-inflammatory effect of probiotics. The cytokine assay results were in complete agreement with the previous in vivo results. The use of *Lactobacillus/Bifidobacterium* before, after, and during inflammation induction was able to significantly decrease the production of IL-6 and IL-1*β*. Molecular study was also consistent with these phenotypic results. The results of JAK/STAT and NF-kB were remarkable. The general trend of all genes examined in the present study after *Lactobacillus/Bifidobacterium* treatment was downward. As mentioned above, the association of the JAK/STATs and NF-kB with various cytokines makes these pathways a suitable target for reducing inflammation. According to Flamant et al., some pro-inflammatory cytokines, including IL-6, IL-9, and IL-12, correlate with severity and disease activity in patients with UC and CD, and STAT1/STAT5, JAK1, JAK3, and TYK2 are associated with these inflammatory cytokines. Therefore, any agent that targets the activation of this pathway and inhibits it could be suitable for the treatment of IBD [20]. Zundler et al. have also shown that STATs may play different roles in the development of IBD. For example, they demonstrated that upregulation of STAT1 is a primary defect that leads to the initiation of inflammation in patients with IBD. They also showed that the deletion of some STATs, including STAT4, may protect patients from colitis progression. In addition, the activation of STAT6 has been shown to lead to a reduction in the induction of Tregs, and thus it plays a critical role in the development of colitis in mice [21]. Similar findings have been reported about JAKs. Leonard et al. have been reported that JAKs are associated with various inflammatory cytokines; therefore, several JAK inhibitors are suitable agents to control and treat IBD [22]. On the other hand, targeting NF-kB is also a suitable way to control IBD, as colitis is associated with the excessive activation of the NF-kB pathway [23]. Many of the therapeutic agents used in IBD, including sulfasalazine, infliximab, and anti-TNF-*α*, have inhibitory effects on the NF-kB pathway [24].

## 5. Conclusion

As mentioned above, there are several treatment options, including monotherapy with chemical drugs, the use of immunomodulators, or surgical treatments for severe forms of the disease [25]. The present study demonstrated the beneficial properties of probiotics in controlling inflammation in both phenotypic and molecular assays. Although phenotypic tests and molecular analysis could show the anti-inflammatory effects of probiotic strains in all treatment phases, the results of molecular studies showed that the use of *Lactobacillus/Bifidobacterium* seems to have a stronger reduction effect after inflammation induction, as it was able to reduce the expression level towards zero in some genes, including *STAT6*, *TYK2*, and *TIRAP*. Indeed, using eight different probiotic strains might be a cause of such a reduction effect. Therefore, the use of probiotics to control and manage symptoms in patients with IBD, especially those in the relapsing phase and active stage of the disease, would be crucial to improve patients' lifestyle. It should be noted that our study has some potential limitations. The use of protein assays such as western blotting (immunoblotting) and thorough evaluation of anti-inflammatory cytokines along with JAK/STAT inhibitors/agonists need to be thoroughly explored.

## Figures and Tables

**Table 1:** Primer sequences used in this study.

Gene	Primer sequence [5′ > 3′]	Primer-bank ID	Product size [bp]
STAT1 F STAT1 R	CGGCTGAATTTCGGCACCT CAGTAACGATGAGAGGACCCT	189458859c3	81
STAT2 F STAT2 R	CTGCTAGGCCGATTAACTACCC TCTGATGCAGGCTTTTTGCTG	291219923c3	87
STAT3 F STAT3 R	ACCAGCAGTATAGCCGCTTC GCCACAATCCGGGCAATCT	47080104c2	124
STAT4 F STAT4 R	GCTTAACAGCCTCGATTTCAAGA GAGCATGGTGTTCATTAACAGGT	345110659c2	91
STAT5 F STAT5 R	CGACGGGACCTTCTTGTTG GTTCCGGGGAGTCAAACTTCC	221316717c3	80
STAT6 F STAT6 R	CGAGTAGGGGAGATCCACCTT GCAGGAGTTTCTATCAAGCTGTG	296010867c2	92
JAK1 F JAK1 R	CTTTGCCCTGTATGACGAGAAC ACCTCATCCGGTAGTGGAGC	102469033c1	101
JAK2 F JAK2 R	ATCCACCCAACCATGTCTTCC ATTCCATGCCGATAGGCTCTG	223671934c2	121
JAK3 F JAK3 R	CTGCACGTAGATGGGGTGG CACGATCAGGTTGGACTTTTCT	189095272c2	78
TYK2 F TYK2 R	GAGATGCAAGCCTGATGCTAT GGTTCCCGAGGATTCATGCC	187608614c1	76
RIP2 F RIP2 R	GCCCTTGGTGTAAATTACCTGC GGACATCATGCGCCACTTT	93141034c2	138
NEMO F NEMO R	AAGAGCCAACTGTGTGAGATG TTCGCCCAGTACGTCCTGA	142381344c1	69
TIRAP F TIRAP R	GACCCCTGGTGCAAGTACC CGACGTAGTACATGAATCGGAG	89111123c2	133
IRAK4 F IRAK4 R	CTTGGATGGTACTCCACCACT AAAATTGATGCCATTAGCTGCAC	223671887c3	76
Gapdh F Gapdh R	GGA GCG AGA TCC CTC CAA AAT GGC TGT TGT CAT ACT TCT CAT GG	378404907c1	197

## Data Availability

The datasets generated during and/or analyzed during the current study are available from the corresponding author on reasonable request.
